# O029: Reporting and case management of bloodborne pathogen exposures among health care workers in Tanzania

**DOI:** 10.1186/2047-2994-2-S1-O29

**Published:** 2013-06-20

**Authors:** M Lahuerta, D Selenic, G Mwakitosha, J Hokororo, H Ngonyani, G Kassa, R Mbatia, SV Basavaraju, C Courtenay-Quirk, Y Liu, D Simbeye, K Kazura, G Antelman, N Bock

**Affiliations:** 1 ICAP-Columbia University, Mailman School of Public Health, USA; 2 Department of Epidemiology, Mailman School of Public Health, Columbia University, New York, USA; 3 Divisions of Global HIV/AIDS, Centers for Disease Control and Prevention , Atlanta, USA; 4 Ministry of Health and Social Welfare, Dar es Salaam, Tanzania, United Republic of; 5 Tanzania Health Promotion Support (THPS), Dar es Salaam, Tanzania, United Republic of; 6 US Centers for Disease Control and Prevention, Dar es Salaam, Tanzania, United Republic of

## Introduction

In sub-Saharan Africa, bloodborne pathogens exposure (BPE) is a serious risk to health care workers (HCW). Reporting BPE is necessary for effective post-exposure prophylaxis (PEP), an important element of workplace safety in health facilities. Limited data are available on factors associated with BPE reporting among HCW.

## Methods

We conducted a cross-sectional study assessing experiences of occupational BPE, history of BPE reporting, and use of PEP among health care workers at three public hospitals in Tanzania. From August to November 2012, HCW were interviewed using Audio-Computer Assisted Self-Interview. All HCW at risk for BPE were invited to participate. Factors associated with reporting BPE were identified using logistic regression.

## Results

Of the 1,102 eligible HCW, 973 (88%) completed the interview. Of these, 690 (71%) were female and 387 (40%) were nurses. Of 357 HCW who had a BPE in the past 6 months, 120 (34%) reported it. Among these 120 reported exposures, 93 (78%) HCW reported within 2 hours of exposure, 98 (82%) received pre- and post-HIV test counseling, and 70 (58%) were offered PEP; 68 (97%) of these 70 HCWs completed PEP. Independent risk factors associated with reporting BPE were being female (adjusted odds ratio (AOR)=2.0 [95% confidence interval (CI) 1.2-3.5), having ever-received BPE training (AOR=2.0, CI 1.2-3.5), knowledge that HCW receive PEP at another facility (AOR=2.6, CI 1.5-4.4) and HIV testing within the past year (AOR=2.3, CI 1.2-4.4).

## Conclusion

Despite the significant proportion of HCW with a recent BPE, only one in three reported it. Our results highlight the importance of appropriate and continuous training on the prevention and reporting of occupational exposures to increase acceptance of HIV testing after BPE.

## Disclosure of interest

None declared.

